# Bacterial Toxins as Pathogen Weapons Against Phagocytes

**DOI:** 10.3389/fmicb.2016.00042

**Published:** 2016-02-01

**Authors:** Ana do Vale, Didier Cabanes, Sandra Sousa

**Affiliations:** ^1^Host Interaction and Response, Instituto de Investigação e Inovação em Saúde, Universidade do PortoPorto, Portugal; ^2^Group of Fish Immunology and Vaccinology, Instituto de Biologia Molecular e Celular, Universidade do PortoPorto, Portugal; ^3^Group of Molecular Microbiology, Instituto de Biologia Molecular e Celular, Universidade do PortoPorto, Portugal

**Keywords:** bacterial exotoxin, macrophage, neutrophil, phagocyte, immunomodulation, innate immune response

## Abstract

Bacterial toxins are virulence factors that manipulate host cell functions and take over the control of vital processes of living organisms to favor microbial infection. Some toxins directly target innate immune cells, thereby annihilating a major branch of the host immune response. In this review we will focus on bacterial toxins that act from the extracellular milieu and hinder the function of macrophages and neutrophils. In particular, we will concentrate on toxins from Gram-positive and Gram-negative bacteria that manipulate cell signaling or induce cell death by either imposing direct damage to the host cells cytoplasmic membrane or enzymatically modifying key eukaryotic targets. Outcomes regarding pathogen dissemination, host damage and disease progression will be discussed.

## Introduction

Macrophages and neutrophils are central mediators of the innate immune system that act at early stages of bacterial infection and have the ability to clear the pathogen through phagocytosis and subsequent digestion (Mantovani et al., 2011; Wynn et al., 2013). Besides their overlapping functional properties, macrophages, and neutrophils also display distinct and specialized features allowing their concerted and cooperative action against pathogens (Appelberg, 2007; Silva, 2010; Silva and Correia-Neves, 2012). Accordingly to their antimicrobial capacity, toxicity, and lifespan, macrophages and neutrophils have different localizations. Long-lived macrophages are distributed in tissues throughout the body where they perform local immune surveillance activities, including recognition, phagocytosis, and rapid signaling of invading pathogens. The number of resident macrophages in resting tissues is rather low. Upon infection resident macrophages secrete chemokines, rapidly recruiting monocytes, and quiescent neutrophils from blood and bone marrow pools to the infectious foci. Monocytes differentiate to short-lived mature macrophages and neutrophils are activated to produce powerful antimicrobial molecules and release proinflammatory cytokines and chemokines amplifying the initial chemotactic role of resident macrophages and sustaining antimicrobial activities (Mantovani et al., 2011; Wynn et al., 2013).

The outcome of an infection is dictated by the nature of host-pathogen interactions and greatly depends on the efficacy of phagocytes. When, through the action of virulence determinants, the pathogen takes the control of the interaction in detriment of the host, the infection establishes. By the contrary, if the host immune defenses dominate over the pathogen, the infection is controlled and the pathogen is eliminated. In this scenario, several human pathogens evolved an arsenal of sophisticated mechanisms to evade host defenses. In particular, given the central role of macrophages and neutrophils as primary phagocytes, several pathogens deploy strategies to either survive or annihilate phagocytes antimicrobial functions (Flannagan et al., 2009). To avoid macrophage antimicrobial properties, some intracellular pathogens interfere with the classical maturation of the phagosome, blocking fusion with endosomes, and lysosomes thereby preventing destruction. Others escape the phagosome to multiply in the cytosol and modulate host gene expression to limit production of cytokines that would be deleterious for pathogen progression. Despite the mechanisms of evasion of neutrophil-killing are yet poorly defined, it is known that some pathogens survive and multiply inside neutrophils which may act as Trojan horses for microbial dissemination (Appelberg, 2007). From the extracellular milieu, some pathogens target macrophage and neutrophils mainly through the secretion of toxins that: (1) cause irreversible damage leading to phagocyte death or (2) heavily perturb intracellular signaling pathways, blocking phagocytosis or modulating inflammation (e.g., through the control of expression of chemokines and cytokines; Lemichez and Barbieri, 2013).

Toxins are potent molecules produced by a large variety of bacterial pathogens that target host cells and play key roles in the host–pathogen dialog. They are major virulence factors often sufficient to determine the outcome of the infection. Indeed, attesting their importance in pathogenesis, the injection of small amounts of some purified toxins can recapitulate many key symptoms of the disease. Bacterial toxins can be divided in several groups regarding their nature and mode of action (Lemichez and Barbieri, 2013). In this review we focus on bacterial exotoxins, which are secreted by the pathogen and act on the host cells from the extracellular milieu. Although exotoxins may target different cell types, some specifically target macrophages and neutrophils thus taking the control of innate immune response, providing the pathogen a suitable environment for active proliferation. Interestingly, while the initial steps of phagocytes intoxication are specific for different exotoxins, the ultimate cellular effects leading to the loss of host cell function are often the same. Taking this into account we review here the mechanisms of phagocyte targeting by archetypal exotoxins such as pertussis toxin (PT) and adenylate cyclase toxin (ACT) secreted by *Bordetella pertussis*, anthrax toxin from *Bacillus anthracis* and *Staphylococcus aureus* leukotoxins. We also include here AIP56, a recently described toxin from *Photobacterium damselae piscicida* (Phdp). In addition, the mode of action of mycolactone, a polyketide molecule produced by *Mycobacterium ulcerans*, and other bacterial secreted products not formally termed as toxins, such as *S. aureus* superantigens-like proteins (SSLs) and phenol-soluble modulins (PSMs), are also reviewed here. Clostridial C3 toxins, which target and modulate macrophage functions, are the focus of another review in this Topic (Barth et al., 2015).

## *Bordetella pertussis*: Two Toxins Are Better Than One

*Bordetella pertussis* is a Gram-negative pathogen that infects the human respiratory tract causing whooping cough, an acute and highly contagious infection (Mattoo and Cherry, 2005; Melvin et al., 2014). Initially thought to be a toxin-mediated disease (Pittman, 1984), such as cholera and diphtheria, pertussis disease is instead the result of the coordinated action of a variety of bacterial factors that allow bacterial adherence to ciliated respiratory epithelium, survival to host innate immune defense, multiplication, and resistance to inflammatory cells (Carbonetti, 2007). Two of the major virulence factors of *B. pertussis* are the secreted toxins, PT and ACT, which emerged as key elements for suppression/modulation of the host immune and inflammatory responses (Carbonetti, 2010; Higgs et al., 2012; Melvin et al., 2014). Interestingly, mouse infections with different mutants suggested that these two toxins have complementary functions in pathogenesis assaulting the innate immune cells at different times and from different angles. PT would act at early stages of infection mainly inhibiting the recruitment of immune cells, while ACT would later intoxicate macrophages and neutrophils blocking bacterial engulfment and destruction (Carbonetti et al., 2005).

### First Round: PT Inflicts First Blow to the Host

**FIGURE 1:**
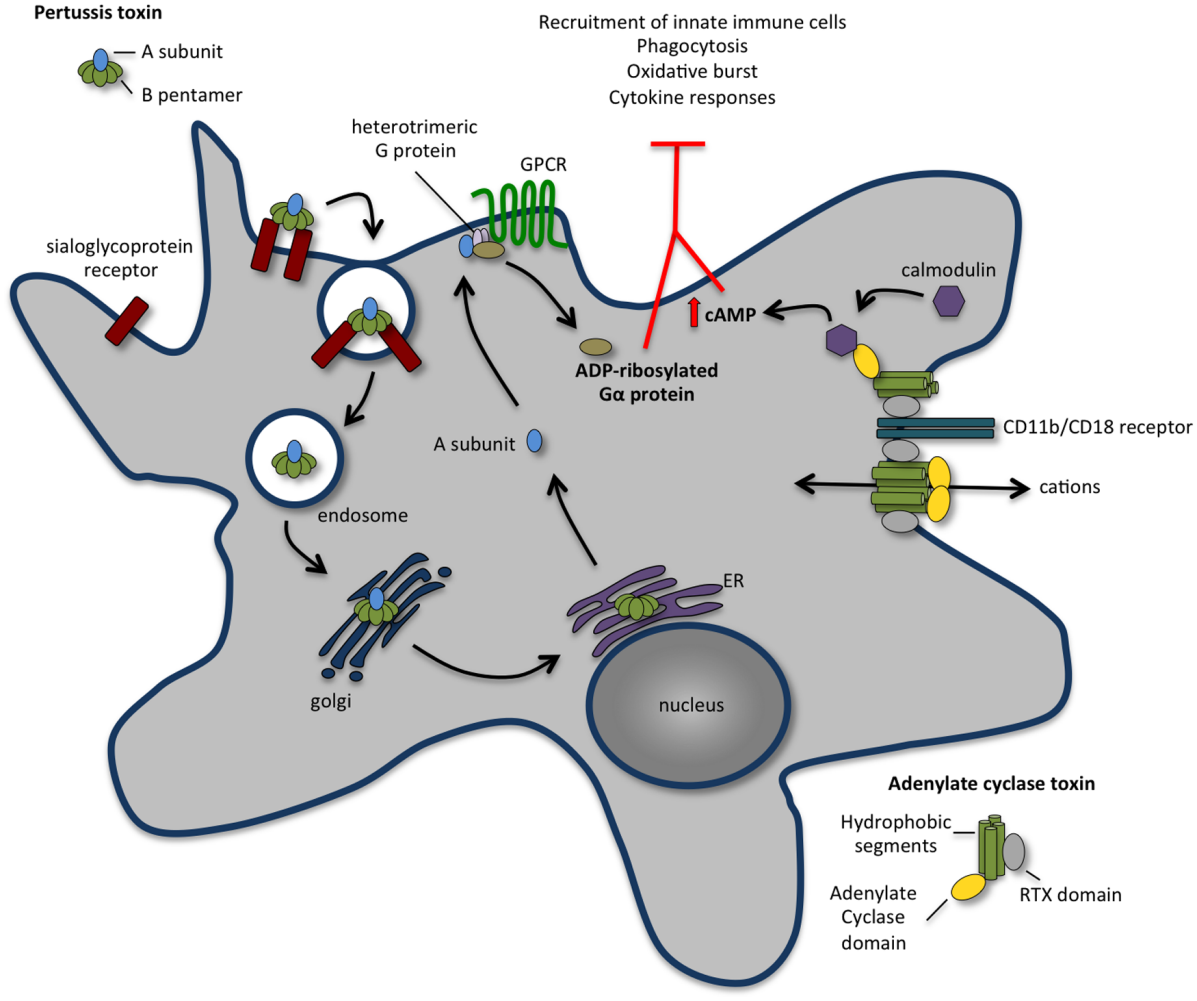
**The concerted action of pertussis toxin (PT) and adenylate cyclase toxin (ACT) annihilates recruitment and function of innate immune cells.** Following binding to a sialoglyprotein receptor, PT is endocytosed and retrogradely transported to the endoplasmic reticulum (ER). From the ER, the A subunit is delivered into the cytosol and travels to the plasma membrane, where it ADP-ribosylates the alpha-subunit of heterotrimeric G proteins, perturbing their regulatory functions and leading to an increase in the cAMP concentration that contributes to the early suppression of inflammatory cytokine production and inhibits the recruitment of immune cells to the site of infection. ACT binds with high affinity to CD11b/CD18 receptor [also known as complement receptor 3 (CR3) or macrophage-1 antigen (Mac-1)] present at the surface of macrophages, neutrophils, and dendritic cells. Upon binding, ACT integrates the membrane of target cell in two different conformations: a translocation precursor that relocalises at lipid raft domains from where the adenylate cyclase activity translocates directly to the cell cytoplasm; and a pore precursor that oligomerises and permeabilises the cells causing ion concentration imbalance. Binding of calmodulin stimulates the adenylate cyclase, leading to an increase in intracellular levels of cAMP. The activity of ACT inhibits complement-mediated phagocytosis, inhibits the production of pro-inflammatory cytokines and interferes with immune cell recruitment.

Pertussis toxin is a multisubunit AB-toxin exclusively produced by *B. pertussis*. Through B-subunits, PT binds to any sialic acid-containing glycoprotein at the cellular surface (Witvliet et al., 1989; Saukkonen et al., 1992; Stein et al., 1994), is internalized by endocytosis and follows a retrograde transport pathway through the Golgi complex to the endoplasmic reticulum (Plaut and Carbonetti, 2008; **Figure 1**), from which the A-subunit translocate to the cytoplasm of host cells (Locht et al., 2011). In the host cell cytosol, the A-subunit exhibits ADP-ribosyltransferase activity, hydrolysing NAD and ADP-ribosylating heteromeric G-proteins of the G_i_ family (Katada et al., 1983). This modification prevents the interaction of G_i_ proteins with their cognate G-protein coupled receptors (GPCRs) causing the disruption of downstream cell signaling transduction (Mangmool and Kurose, 2011). Besides the activity of the A domain, B-subunits also display signaling function by activating intracellular signaling cascades in a G_i_-protein independent manner (Mangmool and Kurose, 2011).

Pertussis toxin has long been considered as a major *B. pertussis* virulence factor. Indeed, PT was reported to be the cause of systemic symptoms of pertussis disease such as lymphocytosis and leukocytosis (Morse and Morse, 1976) and was associated with lethal infection by *B. pertussis* in a neonatal mouse model (Goodwin and Weiss, 1990). However, evidences supporting its role in respiratory infection have only emerged in the last decade. Experiments in the mouse model revealed that the lack of PT confers a defect in *B. pertussis* colonization at the early stages of infection (Carbonetti et al., 2003, 2005). Interestingly, a PT-deficient strain reaches wild type levels of colonization whenever co-infections with both strains are performed or intranasal inoculation of purified PT precedes infection (Carbonetti et al., 2003). Further studies have shown that depletion of resident airway macrophages leads to exacerbated *B. pertussis* infection in a PT-independent manner (Carbonetti et al., 2007), indicating that PT targets airway macrophages disrupting their protective activity at early steps of infection (Carbonetti et al., 2007). Intranasal administration of PT resulted in ADP-ribosylation of airway macrophages G_i_-proteins (Carbonetti et al., 2007), suggesting that its inhibitory function on macrophages *in vivo* results from the immunosuppressive activities ascribed to PT *in vitro*. In particular, *in vitro* PT was shown to inhibit macrophage and neutrophil migration (Meade et al., 1984), phagocytosis (Mork and Hancock, 1993) and cytokine response (He et al., 1988; Hume and Denkins, 1989).

*In vivo*, PT plays a dual role in the establishment of the disease. Whereas it has immunosuppressive functions at early stages of infection, later it potentiates inflammatory responses, likely prolonging healing and promoting bacterial dissemination (Eby et al., 2015). Early in infection, PT targets airway macrophages and inhibits neutrophil recruitment to the infection site (Carbonetti et al., 2003, 2005; Kirimanjeswara et al., 2005; Andreasen and Carbonetti, 2008). Neutrophils were reported to play a protective role against *B. pertussis* only in previously infected mice and in the presence of anti-*B. pertussis* antibodies (Kirimanjeswara et al., 2005; Andreasen and Carbonetti, 2009), which may suggest that PT delays neutrophil recruitment to the airways avoiding rapid antibody-mediated clearance of the pathogen. Later, at the peak of infection, high numbers of neutrophils are recruited to the lungs of mice infected with wild type strain, but not to those infected with PT-deficient strain (Carbonetti et al., 2005; Andreasen et al., 2009). This recruitment of neutrophils to the lungs correlates with an increase of proinflammatory cytokines such as IL-17, TNFα and IFNγ that appears to be dependent on PT activity (Andreasen et al., 2009). Recently, global transcriptional profiles of mice lungs infected with wild type or PT-deficient *B. pertussis* revealed that, at the peak of infection, the ADP-ribosylation activity of PT correlates with upregulation of immune and inflammatory response genes (Connelly et al., 2012). *In vitro* studies suggested that PT directly impairs neutrophil migration through ADP-ribosylation the G_i_-proteins associated with surface chemokine receptors (Spangrude et al., 1985; Scott et al., 1988; **Figure 1**). However, *in vivo* data showed that besides acting directly on chemokine receptors signaling (Kirimanjeswara et al., 2005), PT also suppresses early neutrophil recruitment by inhibiting the production of neutrophil-attracting chemokines by airway macrophages and lung epithelial cells (Andreasen and Carbonetti, 2008). Transcription of genes expressing CXCL1, CXCL2, and CXCL5 is inhibited in the lungs of mice intranasally infected with wild type *B. pertussis*, as compared to animals infected with strains deficient for PT production or producing a PT variant devoid of ADP-ribosylation activity (Andreasen and Carbonetti, 2008). Interestingly, PT also blocks chemokine gene expression and early neutrophil recruitment to the airways following intranasal administration of LPS (Andreasen and Carbonetti, 2008), which occurs presumably through modulation of TLR-4 signaling, a G_i_-protein independent pathway.

Altogether, these studies show that PT suppresses early inflammation in the respiratory tract and inhibit microbicidal function of inflammatory cells, potentially providing advantage to the pathogen by allowing its rapid growth and establishment within the host at early phases of infection.

### Second Round: ACT Perpetuates the Pathogen Gain

Adenylate cyclase toxin is a bi-functional toxin produced by all species of *Bordetella* that infect mammals. While its N-terminal domain contains an adenylate cyclase activity that converts ATP in cAMP, the C-terminus possesses RTX motifs that bind mammalian cells and form cation-selective pores in the host plasma membrane. Through its combined adenylate cyclase and pore-forming activities, ACT manipulates host cell physiology in two different ways: it interferes with intracellular signaling by increasing the levels of cAMP, and disturbs ion homeostasis by disrupting the permeability barrier of the plasma membrane (**Figure 1**). ACT specifically binds with high affinity CD11b/CD18 (also known as CR3 or Mac-1) present at the surface of macrophages, neutrophils and dendritic cells (DCs; Guermonprez et al., 2001). Upon binding, ACT integrates the membrane of target cells in two different conformations: a translocation precursor that re-localizes at lipid raft domains from where the adenylate cyclase activity translocates directly to the cell cytoplasm; and a pore precursor that oligomerises and permeabilises the cells causing ion concentration imbalance (Fiser et al., 2007, 2012; Bumba et al., 2010; **Figure 1**). While the rapid translocation of adenylate cyclase activity across the host plasma membrane does not dependent on endocytosis (Gordon et al., 1988), the clathrin-dependent endocytosis of ACT together with its receptor CD11b/CD18 was reported (Khelef et al., 2001; Martin et al., 2011). Classical mechanisms of membrane repair upon toxin-induced pore formation include the removal of the pores through endocytosis. Interestingly, ACT-translocated molecules control the rate of ACT-pore removal delaying their endocytic uptake, thus exacerbating the permeabilisation of phagocytes and maximizing the cytotoxic action (Fiser et al., 2012). Despite its interaction with a specific receptor, ACT was reported to promiscuously bind and intoxicate many cell types, including CD11b/CD18-negative cells (Ladant and Ullmann, 1999; Paccani et al., 2008; Eby et al., 2012), although the biological relevance of these findings remains unclear. Indeed, while ACT triggers macrophage apoptosis *in vitro* (Khelef et al., 1993; Khelef and Guiso, 1995) and *in vivo* (Gueirard et al., 1998), the viability of cells from non-haematopietic origin remains unaffected (Gueirard et al., 1998; Bassinet et al., 2000). *In vivo* studies support that ACT primarily targets phagocytes such as alveolar macrophages and neutrophils, disrupting the early innate antibacterial host immune response (Harvill et al., 1999; **Figure 1**).

cAMP is a key second messenger with pleiotropic effects. Increased levels of cAMP severely compromise cellular functions such as the migration of neutrophils, the plasticity of DC responses, the release of cytokines by macrophages and the homeostasis of actin cytoskeleton. Thus, through the uncontrolled production of intracellular cAMP, ACT subverts phagocytic, and bactericidal function of macrophages and neutrophils in a variety of ways (**Figure 1**). In human alveolar macrophages and neutrophils, as in mouse macrophages, the ACT-mediated cAMP production blocks phagocytosis, chemotaxis, and oxidative burst (Confer and Eaton, 1982; Friedman et al., 1987; Weingart and Weiss, 2000; Kamanova et al., 2008; Eby et al., 2014). High levels of cAMP cause a transient inactivation of RhoA inducing massive actin rearrangements that dramatically decrease macropinocytosis, block complement-mediated phagocytosis (Kamanova et al., 2008) and possibly impair the chemotactic properties of primary monocytes. Recently, ACT-induced cAMP synthesis was shown to trigger pro-apoptotic signaling in phagocytes through the activity of tyrosine phosphatase SHP-1, the accumulation of cytosolic BimEL and the consequent activation of Bax, permeabilisation of the outer mitochondrial membrane and activation of programmed cell death (Ahmad et al., 2015; Cerny et al., 2015). Besides the immediate action of ACT on the ablation of bactericidal functions of phagocytes, ACT activity was also reported to block the release of TNFα and the production of ROS in human monocytes (Njamkepo et al., 2000), to promote incomplete or aberrant maturation of DCs (Skinner et al., 2004; Boyd et al., 2005) and to impair T-cell activation by interfering with immunological synapse signaling (Paccani et al., 2011). ACT was described to suppress the secretion of pro-inflammatory cytokines such as IL-12 and TNFα and favor the production of anti-inflammatory IL-10 molecules (Spensieri et al., 2006; Hickey et al., 2008).

Despite the fact that studies in mouse models established ACT as an important virulence factor for *B. pertussis* infection (Khelef et al., 1992), the effects described above were obtained from *in vitro* studies, and their significance during *in vivo* infection requires further investigation. Importantly, recent studies using baboon infection model and clinical samples from humans showed that the concentration of ACT in tissues is much lower than the amount used for *in vitro* experiments (Eby et al., 2013), which may compromise the relevance of some effects reported *in vitro*.

Pertussis toxin and ACT have undeniable roles during *B. pertussis* infection and certainly play key functions in the pathophysiology of pertussis disease. Numerous and wide-ranging effects of the purified toxins on cultured cell lines have been reported, however, establishing the correlation of such effects with the human pathology appeared as an incredible difficult task. In addition, the mouse model only provides limited possibilities to address this issue. Together with analysis of clinical samples from humans, the use of baboons as non-human primate model is expected to shed new light on the mechanisms of action of PT and ACT and on pertussis disease.

## *Bacillus anthracis*: Armed To Annihilate The Host Innate Immune Defenses

*Bacillus anthracis* is a Gram-positive, spore-forming rod that causes anthrax, an acute and fast progressing disease that affects humans and other animals, and results from a combination of bacterial infection and toxemia (Moayeri et al., 2015). The spores, which are the infectious form of the pathogen, are able to resist harsh environmental conditions and to infect new hosts when inhaled, ingested, or exposed to skin breaks. Upon entering a potential host, spores germinate into vegetative bacilli that replicate and disseminate through the bloodstream, leading to a systemic infection. Whereas in experimental inhalational anthrax, spore germination requires engulfment by macrophages/DCs (Guidi-Rontani et al., 1999; Hanna and Ireland, 1999), in experimental cutaneous infections it has been shown that most of the spore germination occurs extracellularly (Bischof et al., 2007; Corre et al., 2013). Soon after spore germination, vegetative *B. anthracis* start producing two potent exotoxins – anthrax lethal toxin (LT) and edema toxin (ET) – that along with a poly-D-glutamic acid capsule, are its major virulence factors (Moayeri et al., 2015). *B. anthracis* also secretes anthrolysin O (ALO), a member of the cholesterol-dependent cytolysin family (Shannon et al., 2003). ALO is cytotoxic for several mono- and polymorphonuclear cells (Shannon et al., 2003; Cocklin et al., 2006; Mosser and Rest, 2006) and together with LT, induces apoptosis of macrophages (Shannon et al., 2003). However, ALO-deficient and wild-type strains revealed no differences in virulence in an inhalation infection model (Heffernan et al., 2007). Consistent with these observations, ALO-based vaccines, although conferring protection against lethal intravenous challenge with ALO, do not protect mice against peritoneal infection by *B. anthracis* (Cowan et al., 2007). Altogether, available data indicate that ALO is not essential for *B. anthracis* virulence and thus further studies are needed to determine if ALO contributes to *B. anthracis* pathogenicity.

**FIGURE 2:**
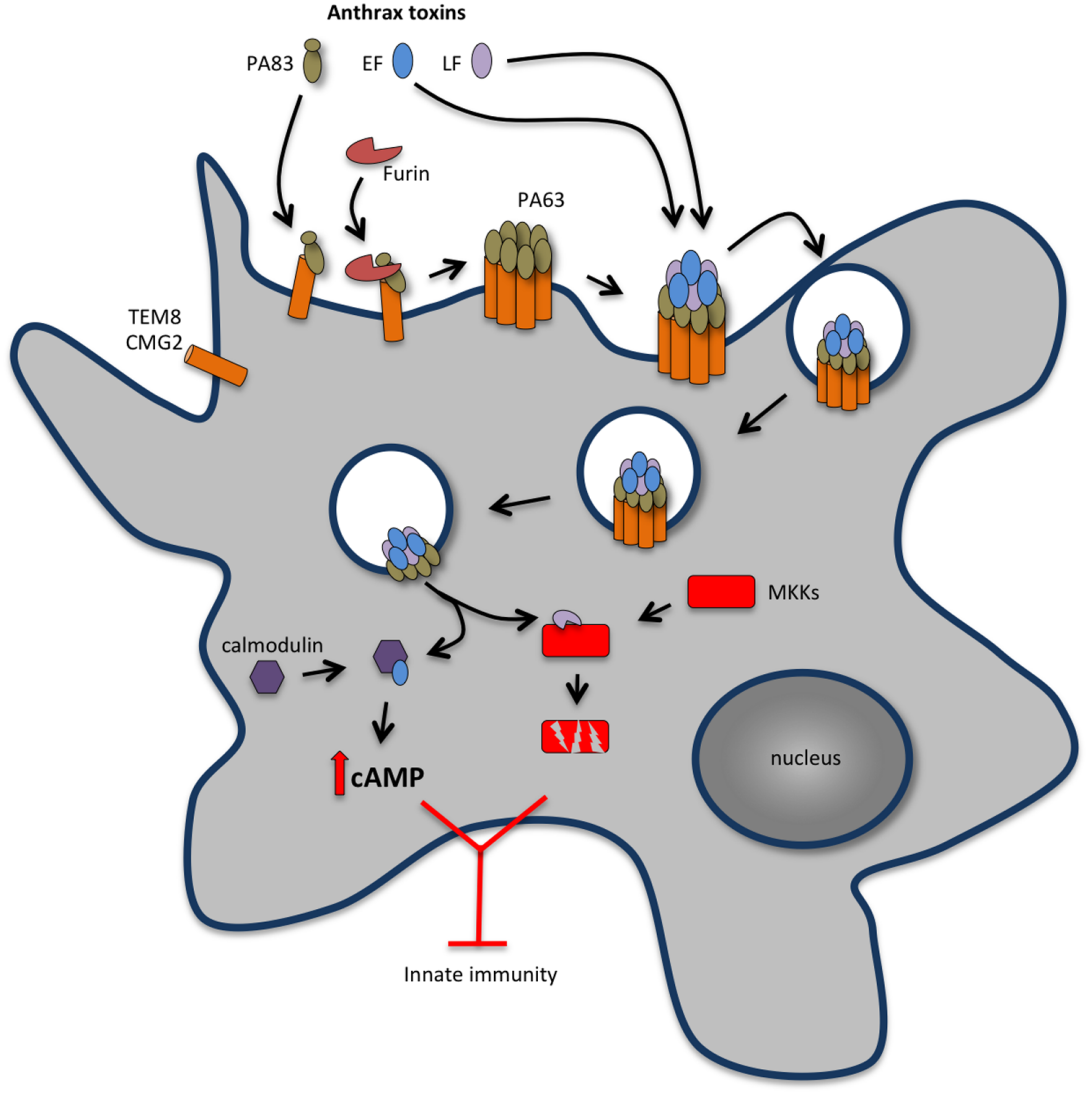
**Anthrax toxins cooperatively disable host innate immune response.** Host cell intoxication by anthrax toxins involves interaction of protective antigen (PA, 83 kDa) with two cellular receptors [tumor endothelium marker 8 (TEM8), also known as anthrax toxin receptor 1 (ANTXR1) and capillary morphogenesis protein 2 (CMG2) also known as anthrax toxin receptor 2 (ANTXR2)], which are expressed by different cell types, including macrophages and neutrophils. Upon binding to the cell surface receptor, PA83 is proteolytically processed at its N-terminus by a furin-like protease yielding the C-terminal fragment PA63 that oligomerises into a heptameric prepore able to bind edema factor (EF) and lethal factor (LF). The EF and/or LF-prepore-receptor complex undergoes receptor-mediated endocytosis and the acidic conditions in endosomes induce conversion of the prepore to a pore, allowing translocation of EF and LF into the cell cytosol to exert their cytotoxic effects. LT is a zinc-dependent metalloprotease that inhibits activation of neutrophils and macrophages, expression of inflammatory cytokines and cell motility by disrupting mitogen activated protein kinase kinases (MAPKKs)-regulated pathways. LT activity also promotes macrophage apoptosis by interfering with pro-survival MAPKK dependent pathways. ET is a calcium and calmodulin-dependent adenylate cyclase that increases intracellular cAMP concentration, leading to the suppression of the expression of inflammatory cytokines and cell chemotaxis through protein kinase A (PKA)-dependent pathways. The concerted action of LT and ET blocks the function of phagocytic cells.

Anthrax toxins originate from the association of three different protein components: a host cell receptor binding protein named protective antigen (PA) and two enzymatic proteins, edema factor (EF) and lethal factor (LF) (Young and Collier, 2007; **Figure 2**). EF or LF associated to PA is referred as ET or LT, respectively. Host cell intoxication begins when PA binds to either tumor endothelium marker 8 (TEM8, also known as anthrax toxin receptor 1, ANTXR1) or capillary morphogenesis protein 2 (CMG2, also known as anthrax toxin receptor 2, ANTXR2), which are expressed by different cell types, including macrophages and neutrophils (Bradley et al., 2001; Scobie et al., 2003). Although both TEM8 and CMG2 can function as anthrax toxin receptors, studies with TEM8- and CMG2-null mice have shown that CMG2 is the major anthrax toxin receptor mediating toxin lethality *in vivo* and that TEM8 plays only a minor role in anthrax toxin-associated pathogenesis (Liu et al., 2009). Upon binding to its cell surface receptor, PA is proteolytically processed at its N-terminus by a furin-like protease and self-assembles forming an oligomeric prepore able to bind EF and LF. The EF and/or LF-prepore-receptor complex undergoes receptor-mediated endocytosis (Abrami et al., 2003) and the acidic conditions in endosomes induce conversion of the prepore to a pore, allowing translocation of EF and LF into the cell cytosol (Collier, 2009) to exert their cytotoxic effects (**Figure 2**). In endosomes, the toxin complex can also be sorted into intraluminal vesicles that undergo back fusion with the endosomal membrane allowing sequestered EF and LF to reach the cytosol (Abrami et al., 2004).

The toxicity of LT and ET results from the catalytic activities of LF and EF. LF is a zinc-dependent metalloprotease that cleaves mitogen activate protein kinase (MAPK) kinases (MKK) 1-4 and 6-7, preventing the activation of the ERK1/2, p38 and JNK-pathways (Duesbery et al., 1998; Pellizzari et al., 1999; Vitale et al., 2000; Tournier et al., 2005) and thus interfering with critical signaling pathways involved in host defense (**Figure 2**). EF is a calmodulin- and Ca^2+^-dependent adenylate cyclase that causes a sustained increase of cAMP (Leppla, 1982) by converting ATP to cAMP. As discussed above in the context of the ACT from *B. pertussis*, increased cAMP levels perturb key cellular functions, leading to severe consequences for the host (Leppla, 1982; Firoved et al., 2005).

### *Anthrax toxins*: Weapons to Cripple Host Macrophages and Neutrophils

Anthrax has for long been described as a toxin-mediated disease, mainly because LT and ET can be lethal for experimental animals and anti-toxin immunization is effective in protecting against infection (Kaur et al., 2013). However, it has recently been proposed that extreme bacteraemia and severe sepsis rather than the anthrax toxins *per se* are the cause of anthrax-induced lethality (Coggeshall et al., 2013). Indeed, the fact that anthrax toxins act on multiple tissues simultaneously, due to the ubiquitous expression of the anthrax toxin receptors, complicate untangling their effects on the host and delayed the identification of the key tissue targets responsible for its lethal effects. Recently, using cell-type specific CMG2-null mice and the correspondent cell-type specific CMG2-expressing mice, Liu and colleagues have shown that LT-induced mortality requires targeting of cardiomyocytes and vascular smooth muscle cells, whereas ET-induced lethality relies mainly on targeting hepatocytes (Liu et al., 2013). Using myeloid-specific CMG2-null mice, in which both macrophages and neutrophils are insensitive to LT and ET due to their inability to bind and internalize the toxins, the same authors have also clarified the role of macrophages and other myeloid cells in anthrax toxins induced lethality and in *B. anthracis* infection (Liu et al., 2010). Myeloid-specific CMG2-null mice are fully sensitive to both LT and ET, indicating that lethality does not depend on the targeting of macrophages, neutrophils, and other myeloid cells (Liu et al., 2010). However, they are completely resistant to infection by *B. anthracis*, indicating that the targeting of myeloid cells by anthrax toxins is required for the establishment of a successful infection (Liu et al., 2010). In what concerns the relative contributions of ET and LT to the establishment of *B. anthracis* infection, trials with Sterne strains deleted of PA, LF, or EF suggest that LT plays a more prominent role (Liu et al., 2010).

Available data indicate that LT and ET act in concert to inhibit macrophage activation as well as the activation and recruitment of other immune cells, such as neutrophils, early in infection (Baldari et al., 2006; Tournier et al., 2009; Liu et al., 2014). This favors bacterial escape and multiplication, and contributes to the severe bacteraemia observed in terminal disease. Macrophage activation requires signaling through MAPK cascades, including JNK and p38 pathways, which are central for induction of inflammatory molecules, including cytokines and chemoattractants, as well as Cox-2 and iNOS. LT interrupts MAPK cascades by directly cleaving MAPKK, whereas ET inhibits MAPK-dependent gene expression by interfering with multiple PKA-related pathways (Baldari et al., 2006). Several reports show that LT inhibits the secretion of pro-inflammatory cytokines by macrophages as well as by DCs *in vitro* and *in vivo* (Pellizzari et al., 1999; Erwin et al., 2001; Agrawal et al., 2003; Alileche et al., 2005; Bergman et al., 2005; Brittingham et al., 2005; Tournier et al., 2005; Ribot et al., 2006). ET has been shown to suppress secretion of inflammatory mediators by DCs (Tournier et al., 2005). Whereas LT inhibits IL-10 secretion by these cells, ET inhibits IL12p70 production (Tournier et al., 2005). Interestingly, LT and ET have a cumulative suppressive effect upon TNFα secretion (Tournier et al., 2005). In addition to suppress pro-inflammatory cytokine secretion by macrophages, LT is also able to trigger programmed cell death in these cells *in vitro*. Indeed, it has been reported that LT induced apoptosis of RAW264.7 cells (Popov et al., 2002). Furthermore, although treatment of three different human monocytic cell lines (HL-60, THP-1, and U937) did not result in cell death, upon differentiation into macrophage-like phenotypes, the cells become susceptible to a cell death program that has features of apoptosis but apparently does not requires the activity of effector caspases (Kassam et al., 2005). The apoptogenic activity of LT toward macrophages likely relates to LT-dependent disruption of survival signals triggered by TLR4 and mediated by p38 MAPK (Park et al., 2002, 2005; Hsu et al., 2004) that activates the NF-kB-dependent expression of pro-survival genes. By cleaving the upstream MAPKK MKK3, LT blocks p38 MAPK and NF-kB activation, leading to macrophage apoptosis (Huang et al., 2004; Park et al., 2005).

Lethal toxin has also been shown to induce a rapid and lytic form of caspase-1-dependent cell death, called pyroptosis, in macrophages from specific rat and mice strains. The susceptibility of macrophages to pyroptosis has been linked to polymorphisms of the *Nlrp1b* gene in mice (Boyden and Dietrich, 2006) and of the orthologous *Nlrp1* gene in rat (Newman et al., 2010). The ability of LT to induce macrophage pyroptosis was initially interpreted as a virulence mechanism of *B. anthracis* (Muehlbauer et al., 2007). It was speculated that the destruction of macrophages by LT compromised their role in restricting *B. anthracis* infection and that the cytokine burst associated to LT-induced macrophage lysis contributed to LT-dependent pathological effects by aggravating the vascular damage occurring in anthrax (Muehlbauer et al., 2007). More recently, it has been shown that LT is able to directly cleave mouse Nlrp1b and rat Nlrp1 close to their N-terminus (Hellmich et al., 2012; Levinsohn et al., 2012; Chavarria-Smith and Vance, 2013) resulting in the activation of Nlrp1 inflammasomes in rat and in mice with LT-sensitive macrophages. Ultimately, this leads to caspase-1 activation and pyroptosis accompanied by the release of the inflammatory cytokines IL-1β and IL-18, which induces a strong innate immune response that is protective against *B. anthracis* infection (Moayeri et al., 2010; Terra et al., 2010). Therefore, the current view is that LT-mediated activation of Nlrp1 that leads to inflammasome activation and macrophage pyroptosis is not a virulence mechanism used by *B. anthracis* to promote infection, but rather a protective host-response against anthrax (Chavarria-Smith and Vance, 2015).

Neutrophils play a major role in controlling *B. anthracis* infection and anthrax-toxin mediated neutralization of neutrophil functions is essential for successful infection (Liu et al., 2010). In *vitro* studies suggest that by reducing F-actin formation, LT and ET cooperate to inhibit neutrophil chemotaxis, chemokinesis, and ability to polarize (During et al., 2005, 2007; Szarowicz et al., 2009). It was also reported that neutrophils intoxicated with ET have reduced phagocytic activity (O’Brien et al., 1985). Additionally, LT has been shown to suppress cytokine production by neutrophils *in vitro* (Barson et al., 2008) and LT and ET were found to block neutrophil priming by LPS or muramyl dipeptide, thereby dampening the oxidative burst normally elicited by bacterial products and required for full antimicrobial activity (Wright and Mandell, 1986). In the case of ET, it has been shown that the inhibition of superoxide production results from an impairment of the activation of the neutrophil NADPH oxidase, an effect that likely results from the activity of ET as an adenylate cyclase (Crawford et al., 2006). Although it has been proposed that these effects are due to the ET-induced rise in cAMP leading to phosphorylation (activation) of PKA (Szarowicz et al., 2009), the downstream targets remain to be identified.

It is now unquestionable that anthrax toxins are crucial in anthrax pathogenesis, but their precise roles during human anthrax infections remain to be further clarified. Following the discovery of the anthrax toxins more than half a century ago, a myriad of studies were developed aiming at defining their role in anthrax disease and lethality. However, most of the infection studies were performed in animal models that may not completely reflect the events occurring during human infections. Concerning the data documenting the immunomodulatory effects of the anthrax toxins, most were obtained *in vitro*, often in experimental set-ups that involve the use of purified toxins and do not allow examining intoxication in the context of infection. Therefore, the available data need to be validated in the context of relevant animal models of infections before being extrapolated to the human disease scenario.

## *Photobacterium damselae piscicida*: Killing Two Birds With One Stone

*Photobacterium damselae piscicida* is a Gram-negative extracellular bacterium that causes a systemic and deadly infection with a rapid course and very high mortalities in both wild and cultured marine fish (Romalde, 2002; Barnes et al., 2005). Phdp infections are characterized by the occurrence of generalized bacteraemia and extensive cytopathology with abundant tissue necrosis (do Vale et al., 2007). Infected fish often present whitish tubercle-like lesions of about 0.5 to 3.5 mm in diameter in several internal organs (Tung et al., 1985; Hawke et al., 1987; Noya et al., 1995; Magariños et al., 1996; do Vale et al., 2007), leading to the coining of the disease as fish pseudotuberculosis (Kubota et al., 1970). The lesions consist of accumulations of bacteria and apoptotic and necrotic cell debris (Kubota et al., 1970; Hawke et al., 1987; do Vale et al., 2007).

*Photobacterium damselae piscicida*-associated pathology is triggered by AIP56 (apoptosis inducing protein of 56 kDa), a plasmid-encoded toxin secreted by virulent Phdp strains (do Vale et al., 2005). The toxin is systemically disseminated in infected animals and induces selective apoptotic destruction of macrophages and neutrophils (do Vale et al., 2003, 2005, 2007). The simultaneous destruction of these cell types by AIP56 has two dramatic consequences for the host. On one hand, the drastic reduction of the number of phagocytes impairs the phagocytic defense, favoring pathogen dissemination (do Vale et al., 2007). On the other hand, it compromises the host capacity to clear apoptosing cells, leading to the lysis of the phagocytes by post-apoptotic secondary necrosis with consequent release of their highly cytotoxic tissue-damaging contents (do Vale et al., 2007; Silva et al., 2008).

### AIP56: Spreading the Misery by Killing the Soldiers and Preventing their Burial

**FIGURE 3:**
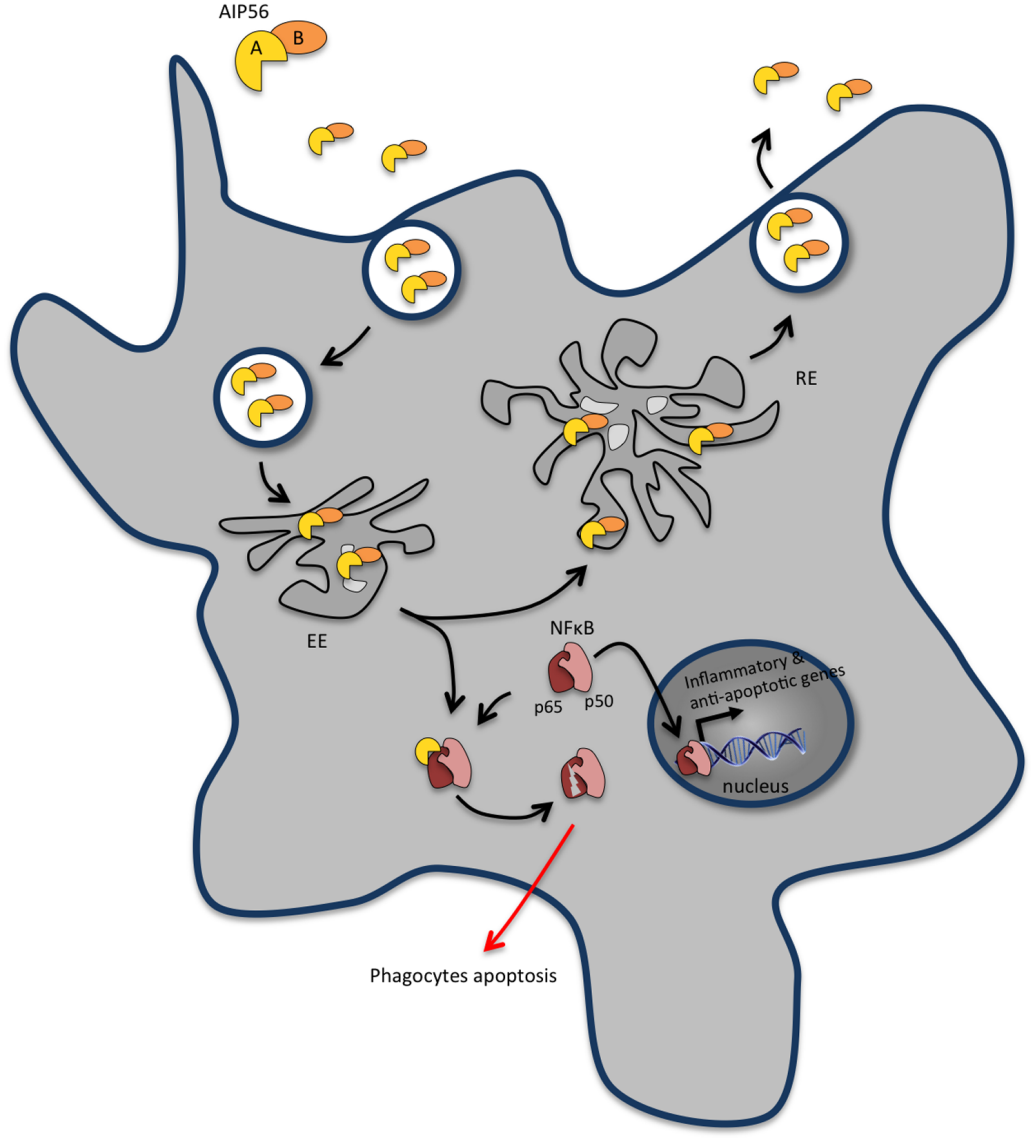
**AIP56 blocks innate immunity by inducing massive apoptosis of host macrophages and neutrophils.** Upon encountering susceptible cells, apoptosis-inducing protein of 56 kDa (AIP56) binds to a still unidentified cell-surface receptor and undergoes clathrin-mediated endocytosis. Once in early endosomes, the toxin either follows the recycling pathway back to the extracellular medium or suffers low pH-induced translocation across the endosomal membrane into the cytosol to display its toxic activity. AIP56 is a zinc-dependent metalloprotease that cleaves the p65 subunit of nuclear factor-κB (NF-κB), an evolutionarily conserved transcription factor that regulates the expression of inflammatory and anti-apoptotic genes and plays a key role in host responses to microbial pathogen invasion. During infection, AIP56 disseminates systemically and its activity leads to depletion of macrophages and neutrophils by post-apoptotic secondary necrosis, thereby blocking the phagocytic defense of the host and contributing to the occurrence of tissue damage.

AIP56 is the founding and the only characterized member of a continuously growing family of bacterial proteins identified in different organisms, mainly marine *Vibrio* species and *Arsenophonus nasoniae*. It is an AB-type toxin, possessing a catalytic A domain at its N-terminal region and a B domain involved in binding/internalization into target cells at its C-terminal region (Silva et al., 2013; **Figure 3**). The catalytic domain of AIP56 is a zinc-dependent metalloprotease that cleaves the p65 subunit of NF-κB (Silva et al., 2013), an evolutionarily conserved transcription factor that regulates the expression of inflammatory and anti-apoptotic genes, playing a key role in host responses to microbial pathogen invasion. AIP56 has likely originated from a fusion of two components: its A domain is related to NleC, a type III secreted effector present in several enteric pathogenic bacteria (Yen et al., 2010; Baruch et al., 2011; Pearson et al., 2011; Sham et al., 2011; Hodgson et al., 2015) that are associated with severe human illness and death worldwide, whereas its B domain is related to a protein of unknown function from the lambda-like bacteriophage APSE2, a phage that infects *Hamiltonella defensa* (Degnan et al., 2009).

Although mammals are not susceptible to Phdp infection, likely due to temperature and osmolality restrictions, AIP56 is able to intoxicate mouse bone marrow derived macrophages (mBMDM; Pereira et al., 2014), through a mechanism similar to the one operating during intoxication of fish cells. Upon encountering susceptible cells, AIP56 binds to a still unidentified cell-surface receptor and is internalized through clathrin-mediated endocytosis (Pereira et al., 2014; **Figure 3**). Once in early endosomes, the toxin either follows the recycling pathway back to the extracellular medium or undergoes low pH-induced translocation across the endosomal membrane into the cytosol to display its toxic activity (Pereira et al., 2014). AIP56 cleaves an evolutionarily conserved peptide bond of the Rel homology domain of NF-κB p65, removing residues crucial for p65-DNA interaction and compromising NF-κB activity (Silva et al., 2013; **Figure 3**). During intoxication, the proteolytic activity of AIP56 results in a complete depletion of p65 and leads to the apoptotic death of cells (Silva et al., 2013; Pereira et al., 2014) through a process involving quick activation of caspases-8, -9 and -3, loss of mitochondrial membrane potential, translocation of cytochrome c to the cytosol and overproduction of ROS (do Vale et al., 2007; Reis et al., 2007a,b, 2010; Costa-Ramos et al., 2011).

AIP56 plays a pivotal role in the establishment of Phdp infection and in the development of the infection-associated pathology. In the initial phase of Phdp infection, when local multiplication of Phdp becomes detectable in infected tissues, extensive infiltration of macrophages and neutrophils occurs (do Vale et al., 2007). As the infection progresses, the pathogen extensively multiplies and disseminates systemically, which leads to a septicemic situation paralleled by the occurrence of AIP56 in the systemic circulation (do Vale et al., 2007). The presence of circulating toxin correlates with the appearance of high numbers of apoptotic macrophages and neutrophils in the peripheral blood, in the spleen, liver, and head–kidney vasculature, as well as in the splenic and head-kidney parenchyma and gut lamina propria (do Vale et al., 2007). This systemic apoptotic destruction of macrophages and neutrophils triggered by AIP56 explains the extensive phagocyte depletion observed in advanced Phdp infections (do Vale et al., 2007). The ability of the toxin to neutralize the main players responsible for the phagocytic defense of the host is a very effective pathogenicity strategy that contributes to the severity of Phdp infections by promoting survival of the pathogen and its unrestricted extracellular multiplication. Concomitantly, the AIP56-induced apoptosis of both professional phagocytes leads to tissue damage with deleterious consequences for the host. In fact, the destruction of macrophages, the cells with the crucial role of eliminating apoptotic cells (Parnaik et al., 2000; Wood et al., 2000), compromises the efficient clearance of apoptotic phagocytes and leads to their lysis by secondary necrosis (do Vale et al., 2003) with release of their cytotoxic intracellular contents (do Vale et al., 2007). This has particularly serious consequences in the case of neutrophils, due to their richness in highly cytotoxic molecules, which damage many cell types and produce tissue injury, thus contributing to the genesis of the Phdp-associated cytopathology.

## *Staphylococcus aureus*: A Bench Of Secreted Molecules To Shoot Neutrophils

*Staphylococcus aureus* is a Gram-positive bacterium that often colonizes the human nares and the skin. Besides being a commensal, *S. aureus* is also a redoubtable human pathogen that causes a variety of severe diseases. To set up a successful infection, *S. aureus* evolved an amazing variety of immune evasive strategies wiping out both innate and adaptive immune responses. *S. aureus* infections involve the invasion of host tissues, replication in abscess lesions and dissemination through purulent drainage of these lesions and require the recruitment of immune cells to the site of infection. Such infiltrated immune cells would usually eliminate the bacteria. However, to counter their action, *S. aureus* secretes soluble molecules targeting multiple pathways to manipulate the capacity of neutrophils for chemotaxis, phagocytosis, and bacterial killing, thus enabling pathogen replication and ensuring the success of the infection. Several recent and comprehensive reviews highlight how *S. aureus* virulence factors manipulate the host immune response (Spaan et al., 2013b; Otto, 2014; Thammavongsa et al., 2015). Here we will focus on its secreted molecules mainly targeting neutrophils, modulating their function, or inducing cell killing.

### Avoiding Neutrophil Extravasation, Chemotaxis, and Activation

**FIGURE 4:**
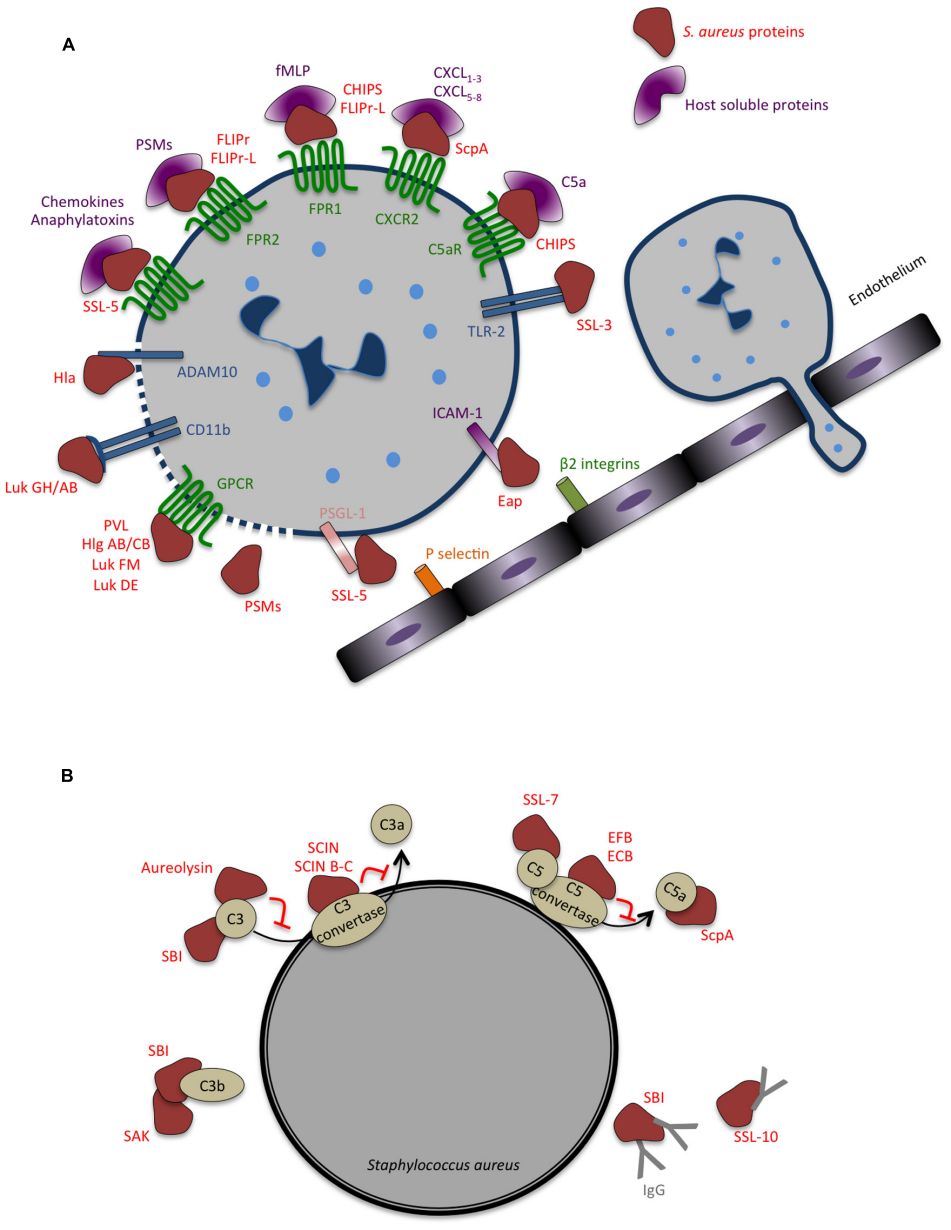
**Strategies evolved by *Staphylococcus aureus* to counteract innate immune response. (A)** Secreted bacterial factors that inhibit neutrophils extravasation, chemotaxis and activation. Neutrophil rolling is modulated by staphylococcal superantigen-like 5 (SSL5) that binds P-selectin glycoprotein ligand-1 (PSGL-1), blocking its interaction with P-selectin. The adhesion of neutrophils to the endothelium and consequent transmigration is inhibited by extracellular adherence protein (Eap), which binds to intercellular adhesion molecule 1 (ICAM-1). In addition to inhibiting PSGL-1, SSL5 inhibits neutrophil responses to chemokines and to anaphylatoxins, by binding to different chemokine receptors. Several staphylococcal molecules impair neutrophil chemotaxis and important co-signaling events during migration and phagocytosis: chemotaxis-inhibitor protein of *S. aureus* (CHIPS) binds and inhibits formyl peptide receptor 1 (FPR1) and C5a receptor (C5aR); formyl peptide receptor-like 1 inhibitor (FLIPr)-like inhibit FPR1; FLIPr and FLIPr-like inhibit FPR2; staphopain (ScpA) cleaves Chemokine (C–X–C Motif) Receptor 2 (CXCR2); staphylococcal SSL3 inhibits toll-like receptor 2 (TLR2)-mediated signaling, the bicomponent leukocidins Panton-Valentine leukocidin (PVL), gamma-hemolysin (Hlg) ABC, leukocidin (Luk) FM, Luk GH/AB, and Luk DE interact with chemoattractant receptors of the G-protein-coupled receptor (GPCR) family. Both Hla and Luk GH/AB induce cell lysis by binding ADAM metallopeptidase domain 10 (ADAM10) and CD11b, respectively. The cytolytic peptides phenol-soluble modulins (PSMs) have an amphipathic alpha-helical region that likely contributes to their lytic activity, presumably by membrane insertion and pore formation. **(B)** Secreted bacterial factors that inhibit opsonization and phagocytosis by neutrophils. The secreted metalloprotease aureolysin inhibits phagocytosis and killing of bacteria by neutrophils by cleaving C3. Staphylococcal complement inhibitor (SCIN), SCIN-B, and SCIN-C associate with and inhibits C3 convertase, thereby preventing the production of C3a, C3b, and further C5a and consequently interfering with complement activation. The extracellular fibrinogen binding protein (EFB) and the extracellular complement-binding protein (ECB) also inhibit complement activation by inactivating C5 convertase and staphylococcal SSL7 targets C5. Staphylococcal binder of immunoglobulin (SBI) affect both the function of complement and immunoglobulin binding, blocking the classical complement activation pathway, and associates with C3 inhibiting the alternative pathway. Staphylokinase (SAK) forms enzymatically active complexes with C3b blocking complement activation. Staphylococcal SSL10 binds IgG, affecting Fc receptor (FcR) recognition and complement activation.

In general, upon pathogen recognition, pro-inflammatory signals released by resident macrophages promote the adhesion of circulating neutrophils and further extravasation across capillary endothelium to the site of infection. This process relies on interactions between the endothelial surface receptors (e.g., selectins and ICAM1) and their respective ligands on the surface of neutrophils (e.g., PSGL1 and β2-integrin) (Spaan et al., 2013b). To inhibit neutrophil recruitment to the infected tissues, *S. aureus* secretes two anti-inflammatory factors that prevent neutrophil adhesion to the blood vessels and further transmigration (**Figure 4A**). The staphylococcal SSL5 binds PSGL1 in a glycan dependent manner at the surface of neutrophils, blocking its interaction with P-selectin expressed by endothelial cells and abrogating the early steps of neutrophil attachment (Bestebroer et al., 2007). In addition, SSL5 was shown to inactivate matrix metalloproteinase from human neutrophils, accounting for the limited capacity of neutrophils to transmigrate into infected tissues (Itoh et al., 2010). The extracellular adherence protein (Eap) recognizes endothelial ICAM1, preventing its interaction with β2-integrins at the surface of neutrophils and further inhibiting extravasation (Chavakis et al., 2002).

*Staphylococcus aureus* also secretes a number of antagonists of neutrophil receptors interfering with chemokine signaling and limiting neutrophil recruitment (**Figure 4A**). In particular, SSL5 directly binds the N-terminus of G-protein coupled chemokine receptors (GPCRs) inhibiting calcium mobilization and actin polymerization, thus impairing neutrophil responses to a huge diversity of chemokines (e.g., CXCL8, CXCL1, CCL2, and CCL5) and to complement fragments C3a and C5a (Bestebroer et al., 2009). Similarly to the recognition of PSGL1 by SSL5, binding to GPCRs relies on the presence of sialic acid residues (Baker et al., 2007; Bestebroer et al., 2007, 2009). In addition, SSL5 was shown to bind to platelet glycoproteins, inducing platelet activation and aggregation, which could be important for colonization and immune evasion by *S. aureus* (de Haas et al., 2009). SSL10 inhibits CXCL12-mediated responses by targeting CXCR4 (Walenkamp et al., 2009) and SSL3 binds to TLR2, hindering immune recognition of staphylococcal lipoproteins and peptidoglycan (Yokoyama et al., 2012).

Phenol-soluble modulins are produced by all *S. aureus* strains and have multiple roles in staphylococcal pathogenesis (Cheung et al., 2014a). In particular, PSMs are potent pro-inflammatory molecules that interact with human formyl peptide receptor 2 (FPR2) (Kretschmer et al., 2010), a GPCR involved in the recognition of pathogens. At nanomolar concentrations, PSMs bind to and activate FPRs, with the strongest activation occurring through FPR2, stimulating several FPRs effector functions such as chemotaxis and pro-inflammatory cytokine production (e.g., CXCL8; Fu et al., 2006). However, human isolates of *S. aureus* evolved other strategies to counter neutrophil chemotaxis by directly interfering with FPRs signaling and limiting cytotoxicity while promoting bacterial replication (McCarthy and Lindsay, 2013; Cheung et al., 2014b). Inhibition of FPRs-mediated pro-inflammatory signaling occurs *via* the secretion of the chemotaxis-inhibitor protein of *S. aureus* (CHIPS) (de Haas et al., 2004) and the FPR2/ALS-inhibitory protein (FLIPr) and its homologue FLIPrL (Prat et al., 2006, 2009; **Figure 4A**). While CHIPS binds and inhibits FPR1 and C5aR, FLIPr and FLIPrL block FRP2-mediated signaling, thus avoiding recognition of PSMs secreted by *S. aureus*, impairing pro-inflammatory response and reducing neutrophil recruitment (Haas et al., 2004, 2005; Prat et al., 2006, 2009). Clearly, CHIPS, FLIPr and FLIPrL, and PSMs have opposite effects on FPRs activation. Thus the production/secretion of these bacterial molecules is likely to be under strict control to allow the establishment of the infection and to evade immune recognition.

The repertoire of secreted molecules by *S. aureus* to evade the early steps of immune response also include Stathopain A (ScpA), a cysteine protease that specifically cleaves the N-terminus of human CXCR2 (Laarman et al., 2012), a GPCR responding to several chemokines (e.g., CXCL1-3 and CXCL5-8). Stathopain A inhibits CXCR2-mediated calcium mobilization, migration, intracellular signaling and activation of neutrophils (Laarman et al., 2012), appearing as an important immunomodulatory molecule causing neutrophil unresponsiveness to several chemokines and blocking their recruitment to the site of infection.

### Blocking Complement Activation, Opsonization, and Phagocytosis

*Staphylococcus aureus* display intrinsic physical features (e.g. thick peptidoglycan layer and capsule) that confer resistance to complement-mediated killing and neutrophil phagocytosis. In addition, *S. aureus* secretes a variety of highly specific proteins with complement-modulating functions (**Figure 4B**), thereby delaying the innate immune attack and generating a window of opportunity to replicate and establish within the host (Spaan et al., 2013b; Thammavongsa et al., 2015). Several of these secreted molecules target C3 or C3 convertases, both central molecules in the complement activation cascade. Aureolysin is a metalloproteinase that cleaves C3, generating a modified C3b fragment that is further degraded instead of being covalently linked to the bacterial surface where it would promote the generation of the chemoattractant molecule C5a (Laarman et al., 2011). In addition, this proteinase degrades human antimicrobial peptides highly potent against *S. aureus* (Sieprawska-Lupa et al., 2004). Thus, aureolysin activity promotes infection by blocking the complement cascade impairing bacterial C3b opsonization, phagocytosis, and neutrophil-mediated killing (Sieprawska-Lupa et al., 2004; Laarman et al., 2011). Staphylococcal component inhibitor (SCIN) specifically binds to human C3 convertase and blocks its activity thereby preventing the production of C3a, C3b, and C5a, thus interfering with complement activation and with neutrophil-mediated killing of *S. aureus* (Rooijakkers et al., 2005a, 2009; **Figure 4B**). In addition, *S. aureus* secreted extracellular fibrinogen-binding protein (Efb) together with its homologue extracellular complement-binding protein (Ecb), were shown to inhibit both C3 and C5 convertases (Jongerius et al., 2010a,b, 2007). Other *S. aureus* secreted proteins target both C3 and immunoglobulin binding, thus impairing complement activation and opsonization, this is the case of staphylococcal binder of immunoglobulin (Sbi; Haupt et al., 2008) and staphylokinase (Rooijakkers et al., 2005b).

Besides their role in blocking neutrophil extravasation and chemotaxis (described above), some SSLs also hinder complement activation and phagocytosis. SSL7 binds IgA and complement C5, inhibiting the production of C5a and further phagocytosis and bacterial clearance during *in vivo* infection (Bestebroer et al., 2010). SSL10 binds to IgG1 preventing recognition by Fc Receptors (FcR), thus impairing IgG1 opsonization and phagocytosis (Patel et al., 2010; **Figure 4B**).

### Avoiding NET Bactericidal Activities

In addition to phagocytosis and intracellular killing, neutrophils evolved an alternative defense mechanism to trap extracellular pathogens and prevent their dissemination. This strategy relies on the release of nuclear content together with antimicrobial cytosolic and granular proteins to form neutrophil extracellular traps (NETs), which are scaffolds that act as physical barriers to pathogen progression protecting host tissues from damage (Papayannopoulos and Zychlinsky, 2009). A secreted staphylococcal nuclease (Nuc) has the capacity to degrade NETs thereby allowing *S. aureus* to resist their bactericidal activities *in vitro* as well as in a respiratory tract infection model (Berends et al., 2010). NET degradation by Nuc leads to the production of monophosphate nucleotides that are further converted into deoxyadenosine through the activity of adenosine synthase (AdsA), another *S. aureus* secreted protein. Interestingly, the accumulation of deoxyadenosine generated by AdsA activity promotes the autocleavage of pro-caspase-3, triggering caspase-3-induced apoptosis of infiltrating macrophages (Thammavongsa et al., 2013). Together, staphylococcal Nuc and AdsA act in a concerted mode to promote bacterial survival in *S. aureus* abscesses, by excluding macrophages from the infection foci.

### Neutrophil Killing: The Ultimate Defense

In line with its ability to evade almost every step of the innate immune response, *S. aureus* induce the death of innate immune cells, trough the secretion of PSMs and several other toxins. As mentioned above, PSMs are *S. aureus* secreted molecules with multiples roles in infection (Wang et al., 2007; Cheung et al., 2014a). They trigger inflammatory responses by interacting with FPR2 and display, at higher concentrations, FPR2-independent cytolytic activity likely through membrane insertion and pore formation (Kretschmer et al., 2010; **Figure 4A**). In particular, PSMα peptides are able to trigger the lysis of the phagosome after neutrophil ingestion allowing intracellular bacterial replication, and ultimately are responsible for lysis of neutrophils promoting bacterial survival and escape to the extracellular milieu (Wang et al., 2007; Geiger et al., 2012; Chatterjee et al., 2013; Grosz et al., 2014). *In vitro*, expression levels of PSMs correlate with levels of cytotoxicity (Rasigade et al., 2013). Moreover, mutants deficient for PSMα production are perturbed in biofilm formation (Peschel and Otto, 2013) and attenuated in the mouse bloodstream infection model (Wang et al., 2007). Altogether, these observations strongly suggest that PSMα-triggered effects may play a key role in *in vivo S. aureus* infection.

In addition to PSMs whose cytolytic activity is receptor-independent, *S. aureus* secretes other cytolytic toxins that interact with specific receptors at the surface of eukaryotic cells, oligomerize and form pores inducing cell leakage and ultimately total lysis. *S. aureus*-produced toxins targeting white blood cells belong to the beta-barrel pore-forming toxins and comprise hemolysin-α (Hla, also called α-toxin) and bicompetent leukocidins (Otto, 2014; Thammavongsa et al., 2015; **Figure 4A**). *In vivo* infection studies have shown that Hla is required for several *S. aureus*-associated pathologies, such as pneumonia and severe skin infections (Bubeck Wardenburg et al., 2007; Bubeck Wardenburg and Schneewind, 2008; Kennedy et al., 2010). Hla interacts with high affinity with ADAM10 at the surface of host cells (Wilke and Bubeck Wardenburg, 2010) to damage epithelial, endothelial and immune cells (Berube and Bubeck Wardenburg, 2013; **Figure 4A**). Mice lacking ADAM10 expression in the lung epithelium resist to lethal pneumonia (Becker et al., 2014), whereas animals lacking ADAM10 specifically on myeloid lineage develop exacerbated skin infections (Inoshima et al., 2011). Although these results suggest that the outcome of Hla-mediated effects may dependent on the infected tissue, the role of Hla on tissue-specific innate immunity requires further analysis.

Leukocidins are composed by two distinct and independently secreted subunits that form heteromultimeric pores in the membrane of host myeloid cells (Otto, 2014). *S. aureus* produce different arrays of leukocidins with different species and cell type specificities, which are mainly dictated by their interaction with host GPCRs (Spaan et al., 2013b; Otto, 2014; Thammavongsa et al., 2015; **Figure 4A**). *In vitro* assays with purified proteins, as well as *ex-vivo* infections with *S. aureus*, have shown that leukocidin AB (LukAB, also called LukGH) kills human, but not mouse, neutrophils upon binding to CD11b (DuMont et al., 2013a). In addition, purified LukAB induce the release of NETs (Malachowa et al., 2013) and also promotes the escape from the phagosome in neutrophils, thus enabling *S. aureus* replication (DuMont et al., 2013b). Gamma-hemolysin HlgAB and HlgCB bind chemokine receptors (e.g., CXCR1, CXCR2, and CCR2) and complement receptors (e.g., C5aR), respectively (Spaan et al., 2014), and promote the lysis of human neutrophils and macrophages *in vitro*. LukED, produced by a large majority of clinical isolates of *S. aureus*, triggers the lysis of neutrophils and macrophages of different vertebrates by binding chemokine receptors such as CCR5, CXCR1 and CXCR2 (Alonzo et al., 2013; Reyes-Robles et al., 2013). Importantly, LukED was shown to play a critical role in *S. aureus* systemic infections in mice, by promoting bacterial replication *in vivo* through direct killing of neutrophils (Alonzo et al., 2012). Lastly, Panton-Valentine leukocidin (PVL), which is secreted by a small percentage of *S. aureus* isolates, binds C5aR on neutrophils and macrophages and has a restricted activity toward human and rabbit cells (Spaan et al., 2013a, 2015). Despite the several attempts to evaluate the exact contribution of each of these toxins to *S. aureus*-associated pathologies, their vast species and cell type specificities have rendered this analysis highly challenging. In this context, the data obtained from commonly used animal infection models (e.g., mice and rats) should be interpreted with caution.

## *Mycobacterium ulcerans*: Sabotage Of The Host Immune Response By A Polyketide Toxin

*Mycobacterium ulcerans* is the causative agent of Buruli ulcer, a chronic ulcerative skin disease that usually starts as painless nodules on the limbs that then develop into large ulcers. These can lead to severe scars and local deformities, including disabling contractures, if not treated at early stages (WHO Buruli ulcer fact sheet N° 199, Updated July 2014). Buruli ulcer occurs most frequently in children living in tropical environments, near wetlands. The disease is more common in poor and rural areas of Africa but is also found in South America, Asia and Australia. *Mycobacterium ulcerans* is currently recognized as an environmental pathogen, but its reservoirs and mode of transmission remain doubtful (WHO Buruli ulcer fact sheet N° 199, Updated July 2014). Genetically very close to *M. tuberculosis* and *M. marinum*, *M. ulcerans* is unique among human pathogenic mycobacteria due to the secretion of a lipid toxin, the mycolactone (George et al., 1999; Hong et al., 2008). Mycolactone displays cytotoxic and immunosuppressive activities and is considered the major pathogenicity factor in Buruli ulcer, being essential for *M. ulcerans* virulence, immune modulation, and colonization (George et al., 1999; Coutanceau et al., 2005; Marsollier et al., 2005; Torrado et al., 2007a, 2010; Simmonds et al., 2009; Guenin-Mace et al., 2011).

**FIGURE 5:**
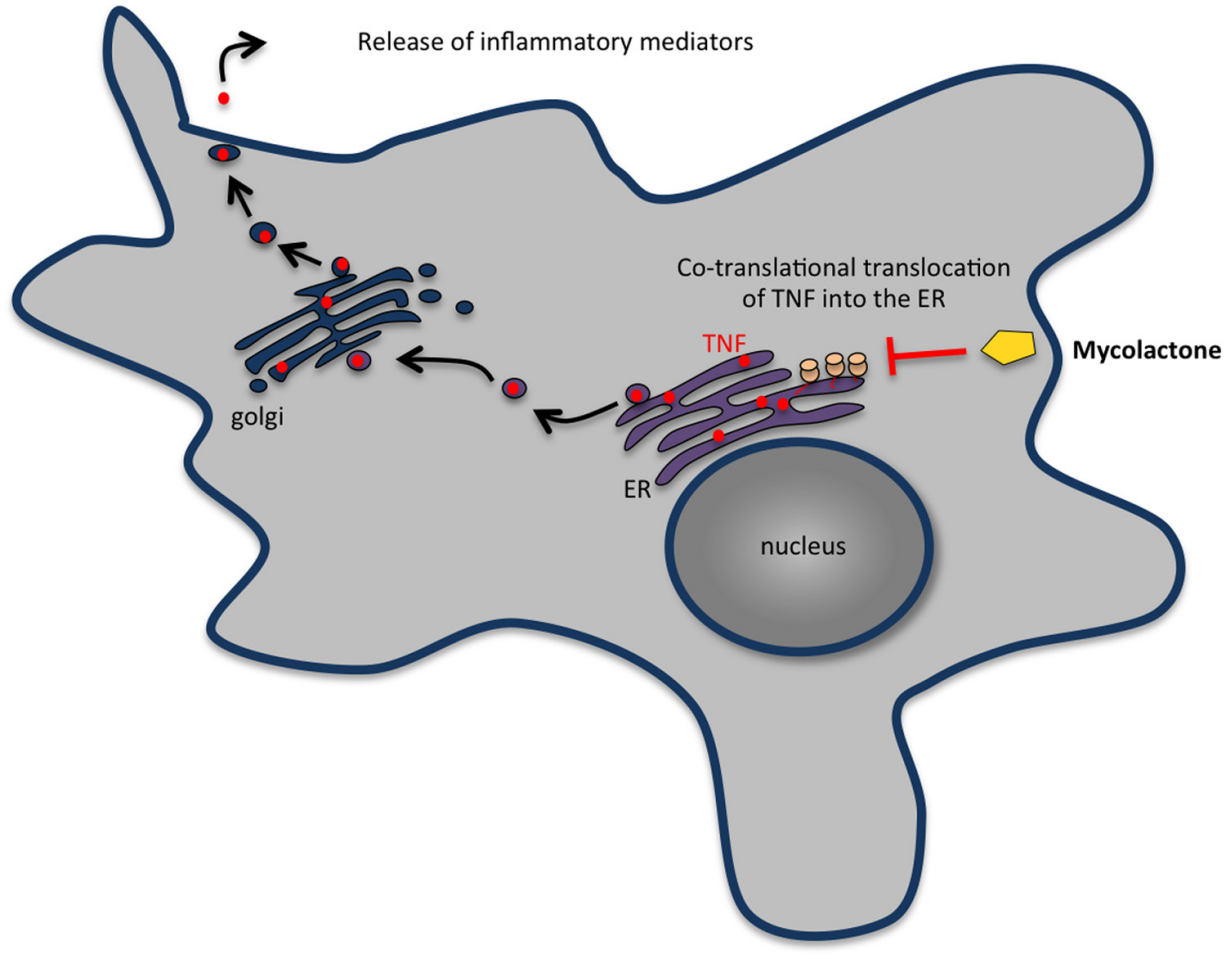
**Mycolactone inhibits the secretion of most cytokines, chemokines and other inflammatory mediators by macrophages.** In eukaryotic cells, secretory proteins cross the ER membrane before being transported in vesicles to the Golgi complex and then to the plasma membrane. Mycolactone enters cells by passive diffusion through the plasma membrane and inhibits the production of inflammatory mediators by macrophages by blocking the translocation of nascent proteins into the ER. The proteins wrongly accumulated in the cytosol are then degraded by the proteasome.

In animal models, injection of mycolactone alone is sufficient to cause ulcers similar to those found in infected hosts (George et al., 1999, 2000). Concerning the mechanism of cellular intoxication, it has been proposed that, due to its hydrophobic nature, mycolactone passively diffuses through the plasma membrane (Snyder and Small, 2003; **Figure 5**). At micromolar concentrations, mycolactone is highly cytotoxic to a variety of mammalian cells, with variable susceptibility levels depending on the cell type (Hall and Simmonds, 2014). The mycolactone cytotoxicity has been linked to its apoptogenic activity. Apoptosis was observed in several cell types incubated *in vitro* with mycolactone (George et al., 2000; Gama et al., 2014), when primary mouse macrophages were incubated with toxigenic *M. ulcerans* strains (Oliveira et al., 2005), and in guinea pigs (George et al., 2000) and mice (Oliveira et al., 2005; Torrado et al., 2007b) infected with mycolactone-producing *M. ulcerans*. More importantly, massive apoptosis has been observed in Buruli ulcer lesions (Walsh et al., 2005). The mycolactone-induced cell death mechanisms appear to be complex and are not completely understood. It has been shown that mycolactone induces cell cycle arrest at the G1/G0-phase (George et al., 1999), but the connection between this effect of mycolactone and its cytotoxicity remains unclear. Early studies on mycolactone reported the occurrence of early actin cytoskeleton rearrangements, cell rounding, and detachment following incubation with the toxin (George et al., 1998, 1999). More recently, studies with HeLa and Jurkat T cells have shown that mycolactone induces increased actin polymerisation in intoxicated cells, as a consequence of its binding to the GTPase domain of the actin-cytoskeleton regulator Wiskott-Aldrich syndrome protein WASP (Guenin-Mace et al., 2013). This leads to hyper-activation of WASP and re-localization of the Arp2/3 complex and consequently, to major cytoskeletal rearrangements, including the formation of filopodia (Guenin-Mace et al., 2013). In epithelial cells, this causes loss of cell adhesion and E-cadherin-dependent tight junctions, ultimately leading to the death of detached cells by anoikis (Guenin-Mace et al., 2013). Recent studies performed *in vitro* with murine fibroblasts confirmed the cytoskeleton as a main target of mycolactone, by showing that mycolactone causes changes in microtubules and affects several regulators and structural components of microtubules and microfilaments (Gama et al., 2014). These deleterious effects inflicted by mycolactone upon the cytoskeleton likely contribute to the formation of the lesions characteristic of Burulli ulcer. Additionally, given the central role of the cytoskeleton in controlling key cellular functions, such as endocytosis, intracellular trafficking, cell adhesion and migration, it is reasonable to speculate that, by highjacking cytoskeleton functions, mycolatone perturbs the functions of phagocytic cells. Indeed, it is likely that the decreased phagocytic activity of macrophages exposed to mycolactone (Adusumilli et al., 2005; Coutanceau et al., 2005) results from the effect of the toxin upon the cytoskeleton of those cells. Further investigations are required to determine whether cytoskeleton is manipulated by mycolactone in macrophages and neutrophils and what are the consequences of this manipulation *in vivo*.

In addition to its known cytotoxic effects toward distinct cell types, at non-cytotoxic concentrations, mycolactone interferes with important functions of immune cells, including monocytes, macrophages, and DCs (Arango Duque and Descoteaux, 2014; Hall and Simmonds, 2014). It is well recognized that macrophages play crucial roles in mycobacterial infections, including in Buruli ulcer. Although Buruli ulcer histopathology is characterized by extensive areas of necrosis with abundant extracellular bacteria, studies on infected humans, and experimental *M. ulcerans* infections revealed that *M. ulcerans* is phagocytosed by macrophages and neutrophils, similarly to the other pathogenic mycobacteria (Torrado et al., 2007b; Silva et al., 2009) and escape the microbicidal activity of the macrophages presumably by mycolactone-dependent interference with the IFNγ–dependent phagosome maturation and NO production required to control *M. ulcerans* infection (Torrado et al., 2010). After an initial phase of intracellular proliferation, varying according to the strain cytotoxicity/virulence, *M. ulcerans* causes apoptosis/necrosis of the host macrophage through a mycolactone-dependent mechanism and becomes extracellular (Torrado et al., 2007b).

Usually, phagocytosis of a microorganism triggers signaling events that rapidly culminate in a controlled inflammatory response involving the secretion of several cytokines and chemokines that recruit other inflammatory cells to the site of infection. However, available evidence suggests that this early response is heavily perturbed by mycolactone. Indeed, cells exposed to mycolactone-producing strains of *M. ulcerans* secrete much less TNF than those infected with mycolactone-negative strains (Torrado et al., 2007a) and purified mycolactone has been shown to supress the production of several cytokines, chemokines, and other inflammatory mediators by macrophages (Arango Duque and Descoteaux, 2014; Hall and Simmonds, 2014). It has been proposed that this suppression is associated with a mycolactone-induced blockade of co-translational protein translocation into the ER and subsequent degradation of the aberrantly located proteins in the cytosol (Hall et al., 2014; **Figure 5**). Failure to produce cytokines and chemokines may contribute to the absence of inflammatory infiltrate at the central necrotic areas of the lesion containing high numbers of extracellular bacilli, in addition to the lysis of recruited inflammatory cells induced by the build-up of mycolactone. The inflammatory infiltrates occupy a band at the periphery of the lesion that represents a front that is continuously advancing into healthy tissues in progressive *M. ulcerans* lesions (Silva et al., 2009).

## Concluding Remarks

The clearance of infectious agents greatly depends on the host innate immune responses that take place at early stages of infection and in which macrophages and neutrophils are the central players. To counteract the host defense mechanisms, bacterial pathogens secrete a bench of different toxins that neutralize, at different levels, the host innate immune response and in particular, annihilate the function of macrophages and neutrophils. Despite having different features, secreted toxins targeting the function of innate immune cells often display similar and/or complementary activities and modulate the same central pathways of the host cell (e.g., inflammatory response, cytoskeleton dynamics, and cAMP signaling). Furthermore, a single pathogen may secrete several toxins that act differently to produce the same outcome (e.g., inhibit chemotaxis or induce phagocyte death). These apparently redundant strategies of bacterial attack ensure the multistep impairment of the early host immune responses mounted against the pathogen and guarantee the control of host–pathogen interaction providing a window of time and opportunity for bacterial growth and establishment within the host.

In the past, many studies aiming to uncover the molecular functions of bacterial toxins on host cells were performed *in vitro* in several cultured cell lines, and more recently in primary cells, using a wide range of concentrations of purified toxins. In addition, studies on animal models (mainly in rodents) using either purified toxins, wild type bacteria and toxin-deficient mutants, provided a number of important observations regarding the toxin-mediated pathologies. Together, these studies generated an incredible amount of data that paradoxically poorly contributed to the understanding of the role of toxins in human infections. Whereas in the perspective of using toxins as molecular tools to address cell biology topics there is great value in testing toxin effects in many *in vitro* cell systems, several issues render extremely difficult the interpretation of data from *in vitro* studies in the context of infection. In particular, the concentration of purified toxin used is often much higher than that produced by bacteria during infection and it is highly variable among different studies, and the cell lines tested are often non-relevant for the pathophysiology of the infection. Regarding studies performed in animal models, two major concerns have been pointed out: (1) many toxins display species-specificity and thus routinely used models, specially rodents, are non-relevant for the study of many toxin-mediated human pathologies compromising the extrapolation of data and (2) direct inoculation of purified toxins in the animals only provide limited information that do not necessarily recapitulate the effect of a given toxin in the context of human bacterial infection. Thus, the data generated so far needs to be cautiously analyzed whenever we aim to better understand the role of toxins in the *in vivo* infectious process. The accurate role of toxins in human infections needs to be analyzed in the context of bacterial infections in different animal species. In this perspective, future efforts should concentrate in the development and use of appropriate animal models, possibly non-human primates, in which available *in vitro* and *in vivo* data can be confirmed and possibly extrapolated to the human pathologies.

## Author Contributions

AdV and SS wrote the manuscript. DC contributed with figures design. The manuscript content was discussed and decided by all the authors.

## Conflict of Interest Statement

The authors declare that the research was conducted in the absence of any commercial or financial relationships that could be construed as a potential conflict of interest.
